# Supplementary material for: Short-range order controlled amphoteric behavior of the Si dopant in Al-rich AlGaN
Source: Nat Commun. 2025 May 30;16:5005. doi: 10.1038/s41467-025-60312-4 (PMC12122704; doi:10.1038/s41467-025-60312-4)
Supplement: Supplementary file 1 — Supplementary Information [file 41467_2025_60312_MOESM1_ESM.pdf]

Supplementary Information

Short-range order controlled amphoteric behavior of the Si dopant in Al-rich AlGaN

Igor Prozheev[1,*], René Bès[1], Ilja Makkonen[1], Frank Mehnke[2], Marcel Schilling[2], Tim Wernicke[2], Michael Kneissl[2,3], and Filip Tuomisto[1]

1 - Department of Physics and Helsinki Institute of Physics, University of Helsinki, P.O. Box 43, FI-00014 Helsinki, Finland.

2 - Institute of Solid State Physics, Technische Universität Berlin, Hardenbergstr. 36, EW 6-1, 10623 Berlin, Germany

3 - Ferdinand-Braun-Institut, Gustav-Kirchhoff-Str. 4, 12489 Berlin, Germany

* Corresponding author: filip.tuomisto@helsinki.fi

I. SUPPLEMENTARY METHODS

Positron annihilation spectroscopy

Supplementary Figure 1 shows the (S,W) parameters as a function of positron implantation energy and corresponding mean implantation depth measured in the Si-doped AlGaN samples H6−H13 at room temperature. The dashed line marks the characteristic (S,W) parameters of the Mg-doped GaN reference, where positrons annihilate in the free state in the GaN lattice.[1] The S parameter decreases from its elevated surface values at energies $0-5$ keV, until it reaches the characteristic values of the Si-doped AlGaN layer of interest in the range $5-15$ keV, corresponding to the thickness of the layers. The S parameter further gradually approaches the characteristic values of the substrate at energies above 15 keV. As is typical, the W parameter behaves in a similar but mirror-like manner to S parameter. The relative (S,W) parameters characterizing the layers and shown in Figure 2 of the main paper are obtained as averages in the implantation energy range that best describes the layer, and normalized to that obtained in the GaN reference.

Supplementary Table I shows the resistivity data, Si concentrations and cation vacancy concentrations estimated from the positron annihilation data for all the studied samples. The carbon

concentrations are also shown even if they are the same for all samples within the series for easy comparison with the cation vacancy concentrations. The data for the samples L1-L5 and H1-H5 are taken from Supplementary Ref. 2. It was shown with temperature-dependence positron experiments in Supplementary Ref. 2 that carbon acts as a negative acceptor ion the positron data, corresponding to its concentration. Similarly as in AlN, the negative ions in Al-rich AlGaN are efficient traps even at roomt temperature,[3] and need to be taken into account when estimating vacancy concentrations. The vacancy concentrations shown in Supplementary Table I are obtained in this way, following the same reasoning as in Supplementary Ref. 2.

The analysis of the temperature-dependent data (see Supplementary Ref. 2) in the case of sample L5 revealed that a negative ion that produces (S,W) parameters of AlN is needed at concentrations $> 5 \times 10^{18}$ cm^{-3} to explain the behavior. We note that in the analysis of the L series samples, where the C concentration is an order of magnitude lower than in the H series samples, the effect of C of the positron data is negligible. Similarly, in the case of sample H13 where C is taken into account in the analysis, an additional negative ion that produces (S,W) parameters of AlN at concentrations close to 1×10^{19} cm^{-3} is needed to analyse the data point in Fig. 2 in the main paper. The S parameter (similarly for the W parameter) is in this case defined as:

$$S = \frac{\lambda_B + \kappa_{\mathrm{C}}}{\lambda_B + \kappa_V + \kappa_{\mathrm{C}} + \kappa_{\mathrm{AlN}}} S_B + \frac{\kappa_{\mathrm{AlN}}}{\lambda_B + \kappa_V + \kappa_{\mathrm{C}} + \kappa_{\mathrm{AlN}}} S_{\mathrm{AlN}} + \frac{\kappa_V}{\lambda_B + \kappa_V + \kappa_{\mathrm{C}} + \kappa_{\mathrm{AlN}}} S_V, \qquad (1)$$

where κ_{C} and κ_{AlN} are the trapping rates to C acceptors and the AlN-like negative ions, respectively, and S_{AlN} is the S parameter of the AlN lattice. After obtaining κ_{AlN} from the analysis, one can remove the effect of C on the measured (S,W) parameters to check where the data point would theoretically be located if there was no C in the sample. This is shown as the shadow marker in Fig. 2 in the main paper. The effect is less pronounced in the case of sample H12 (and L4), but still visible. The high concentration of the AlN-like negative ion causes an additional uncertainty in the concentration estimate of the cation vacancies, the values are hence given in parentheses in the case of these samples.

XANES data treatment

All acquired spectra of incident X-rays (I_0) contained a visible Si K-edge, which was linked to one harmonic suppression mirror on the beamline optic. While a weak Si absorption signal should not be an issue for other experiments performed at LUCIA, such a contribution can be significant in our data due to the low concentration of Si in our samples. To ensure comparability between spectra we checked that all collected I_0 signals were identical. This gave us the opportunity to rule out I_0 artefacts not to be responsible of the observed differences in the XANES spectra and to use the deadtime corrected fluorescence yield data not divided by I_0. We used the Athena software package for normalization and data treatment.[4] Typically, we merged all successful individual scans and normalized the merge by subtracting best fit lines above and below the edge, so that the resulting edge jump is 1.0. Next, we defined E_0 at the maximum of the largest peak in the first derivative for each spectrum, and subtracted the background from the post-edge region. This way we justify a direct comparison of spectra with fingerprinting method for discussing the change in the XANES spectra between the samples.[5]

II. SUPPLEMENTARY DISCUSSION

Electronic structure calculations

Supplementary Figure 2 shows the relative formation energies of (a) Si_{Al} and (b) Si_{Ga}. The effective correlation energy U associated with the formation of DX^- is usually defined as

$$U = E^+ + E^- - 2E^0, \tag{2}$$

where E^+ and E^0 are the formation energies of the donor impurity in the positive and neutral charge states, and E^- is the formation energy in the negatively charged DX configuration.[6] Traditionally, negative U values indicate a stable DX center. However, since we are modelling the formation energies using alloy systems, it would make no sense to calculate all the energies in Eq. (2) using the same Si position and the same model with same configuration of Al and Ga, but one would have to consider how the distribution of formation energies would look like. This would require extensive work, but luckily there is a simpler solution. For a DFT cell with only one Si atom in either neutral, positive or negative DX states, the position of the (+/–) charge-state

transition level in relation to CBM determines the stability between positive and negative charge states of the defect.[7] We find that Si_{Al} forms a DX center in AlN, with $U = -0.13$ eV. The (+/–) transition level occurs at 0.17 eV below the conduction band minimum (CBM). Which is in a good agreement with the previously reported values of $U = -0.30$ eV and transition level at 0.15 eV below the CBM reported for Si_{Al} in Supplementary Ref. 8. Silicon is a shallow donor in GaN and forms stable Si_{Ga}^{+} state for all Fermi levels inside the band gap. We are able to obtain a stable DX configuration for Si_{Ga} in GaN, but the (+/–) transition occurs 1.68 eV above the CBM. Supplementary Reference 8 reports the (+/-) transition level at 1.65 eV above the CBM Si_{Ga} in DX configuration.

In order to study the properties of the Si DX state in AlGaN material, we calculated the partial charge densities attributed to the bands introduced by Si at the Γ point. Supplementary Figure 3 shows atomic structure and the charge density of the DX-induced occupied state in the gap with the isosurfaces of the electron density of states (DOS) set to 5% of the maximum value. The charge is localized in the vicinity of the broken Si-N* bond. Supplementary Figure 4 shows atomic structure and partial electron density of states of the Si atom in negative charge state. In contrast to DX, Si_{GA}^{-} which is a global energy minimum has a delocalized charge all around the supercell.

Random alloy composition

As long as there is no direct evidence of clustering, segregration or ordering, the assumption of the atoms being randomly distributed in an alloy is appropriate. It is, however, important to note that a random distribution is not homogeneous when the scale is brought down to the atomic level. Supplementary Figure 5 shows the probabilities for numbers/fractions of Ga atoms in the vicinity of a cation site, obtained from the binomial distribution in $Al_{0.90}Ga_{0.10}N$. Several observations are evident. On the atomic scale, that is in the 12-atom second-nearest neighbor (SNN) shell, the probabilities for having 0, 1 or 2 Ga atoms are roughly the same (and naturally the highest). However, as seen in the inset, even the probability for 6 Ga atoms in the SNN shell, equivalent locally to 50% Ga content, is not vanishing, being less than 3 orders of magnitude lower than that for 0-2 Ga atoms. This means that the concentration of such cation sites is higher than 10^{19} cm^{-3} in the random alloy. Similarly, when analysing the scale at which electronic structure calculations are usually performed, that is $\sim$200 atoms in the supercell, we see that the probability of encountering a region with less than 2% of Ga, equivalent to 0-2 Ga atoms in the supercell is not vanishing,

either. The order of magnitude is similar as in the previous case, that is, there are more than 10^{19} cm^{-3} cation sites with the 200 surrounding atoms forming a AlN-like 200-atom region in $Al_{0.90}Ga_{0.10}N$. The homogeneity of the atomic distributions increases rapidly with increasing the size of the considered region of a random alloy, as seen for the case of 2000 considered atoms, equivalent to the number of atoms in a roughly 2 nm radius surrounding a cation site. The 10% nominal Ga atomic fraction is in this case $\sim 7-13\%$ if the same limits of relevance are employed.

III. SUPPLEMENTARY TABLES

Supplementary Table I. The measured resistivity and concentrations of Si and C impurities together with the estimated concentrations of the V_{III} vacancies in the MOVPE AlGaN:Si samples.

Sample	Resistivity Ω·cm	[Si] $\times10^{18}$ cm^{-3}	[C] $\times10^{18}$ cm^{-3}	[V_{III}] $\times10^{18}$ cm^{-3}
L1	0.7	0.9		>5
L2	0.54	2.4		>5
L3	0.69	5.0	0.2	>5
L4	1.35	8.5		(>5)
L5	10.82	12		(>5)
H1	>13.4	1.1		<0.01
H2	6.63	2.1		0.2
H3	1.9	3.5		0.6
H4	0.99	5.4		1
H5	0.61	7.0		2
H6	4.5	1.1		0.2
H7	4	2.1	2.0	0.6
H8	1	5.1		1
H9	0.79	7.0		3
H10	0.77	8.8		2
H11	0.8	10		5
H12	0.79	13		(5)
H13	1.82	17		(5)

IV. SUPPLEMENTARY FIGURES

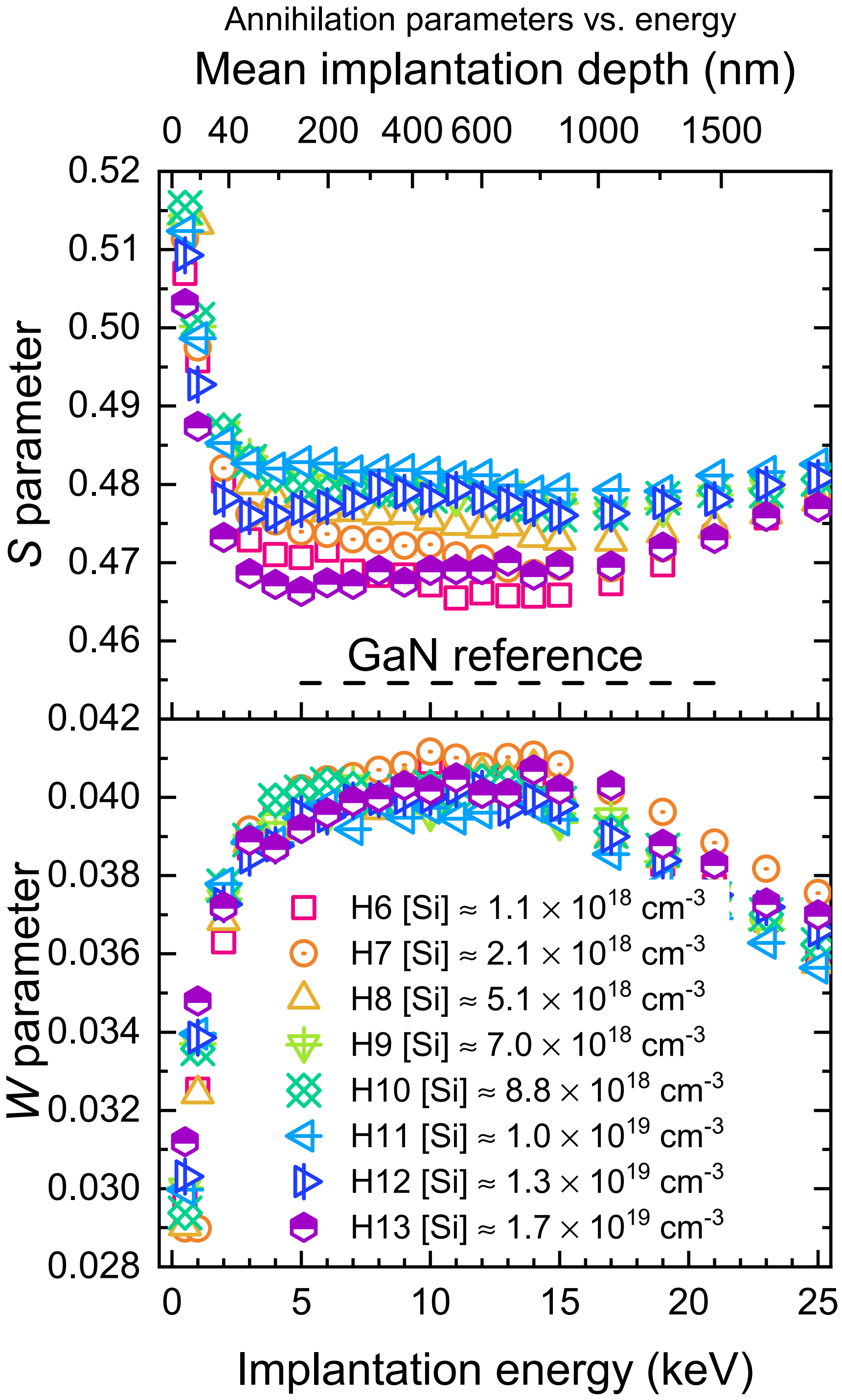

Supplementary Figure 1. The S and W parameters as a function of positron implantation energy and corresponding mean implantation depth in selected Si-doped AlGaN alloys measured at room temperature. The characteristic S parameter of the Mg-doped GaN reference are marked with a dashed line in the upper panel, the corresponding reference W parameter value of 0.455 is not marked for the sake of visual appearance.

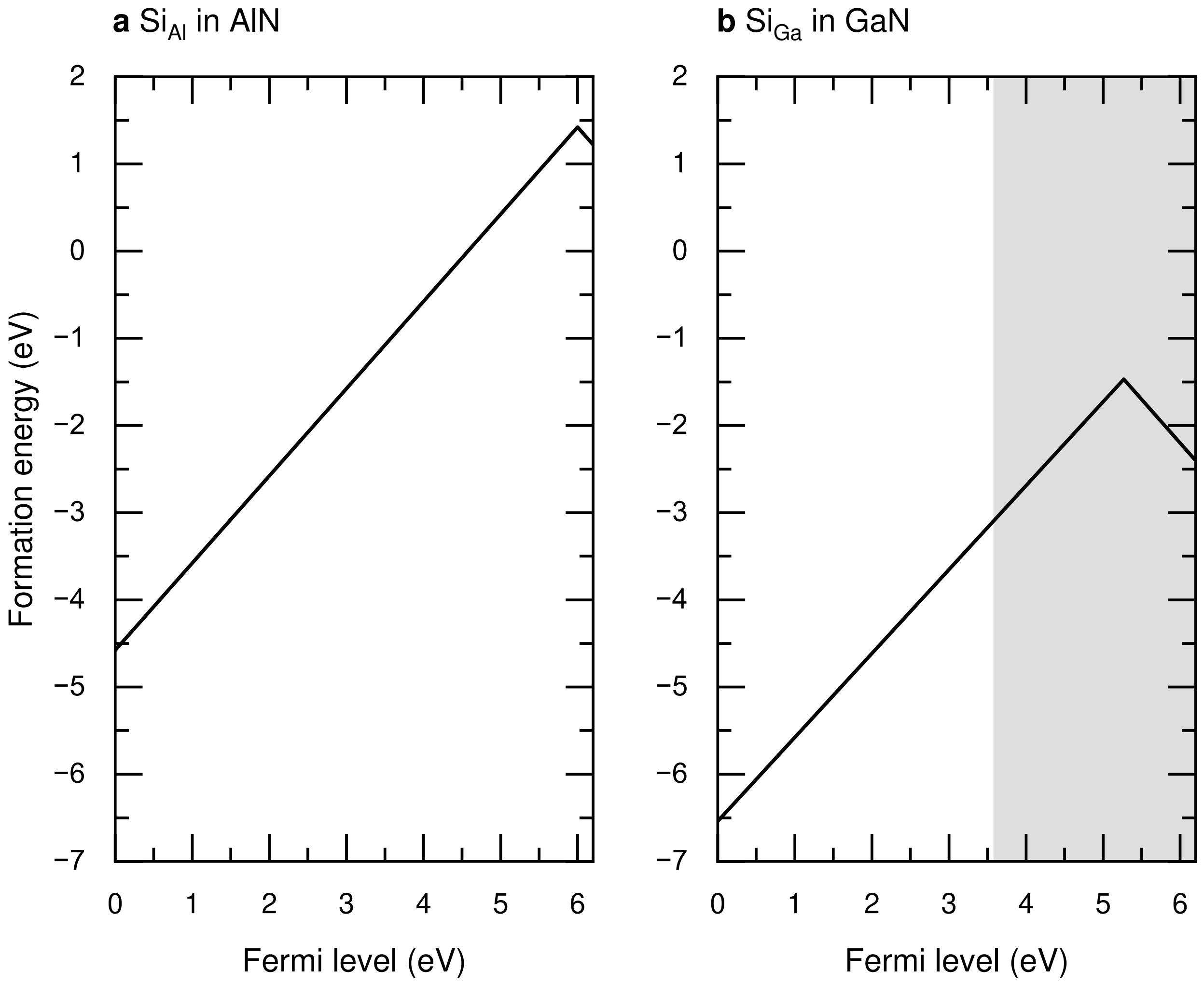

Supplementary Figure 2. Relative formation energy as a function of Fermi level for Si on the cation substitutional site in **a** AlN and **b** GaN. In GaN, Fermi levels above the calculated gap of 3.58 eV are indicated by the shaded area.

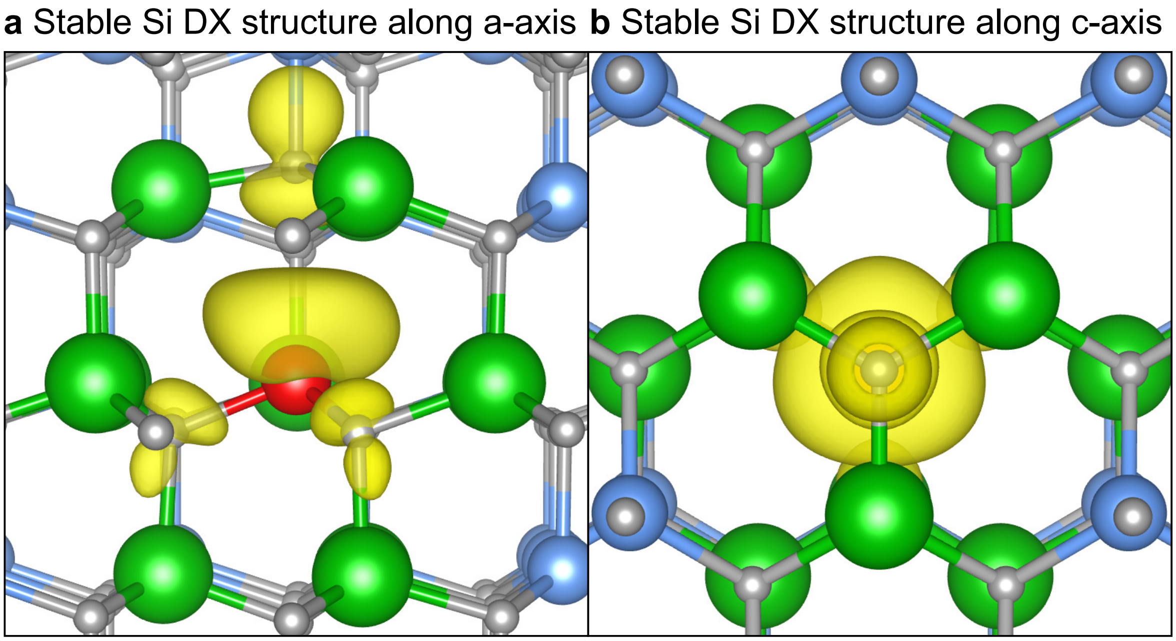

Supplementary Figure 3. Atomic structure and visualized charge density in the stable Si DX configuration along **(a)** a- and **(b)** c-axis. The silicon atom (red sphere) sits on the substitutional site normally occupied by aluminum (blue spheres) inside a cluster formed by 12 gallium atoms (large green spheres); nitrogen atoms are denoted by small gray spheres. Yellow isosurface shows electron density of state which is localized around the broken bond with 5% of the maximum.

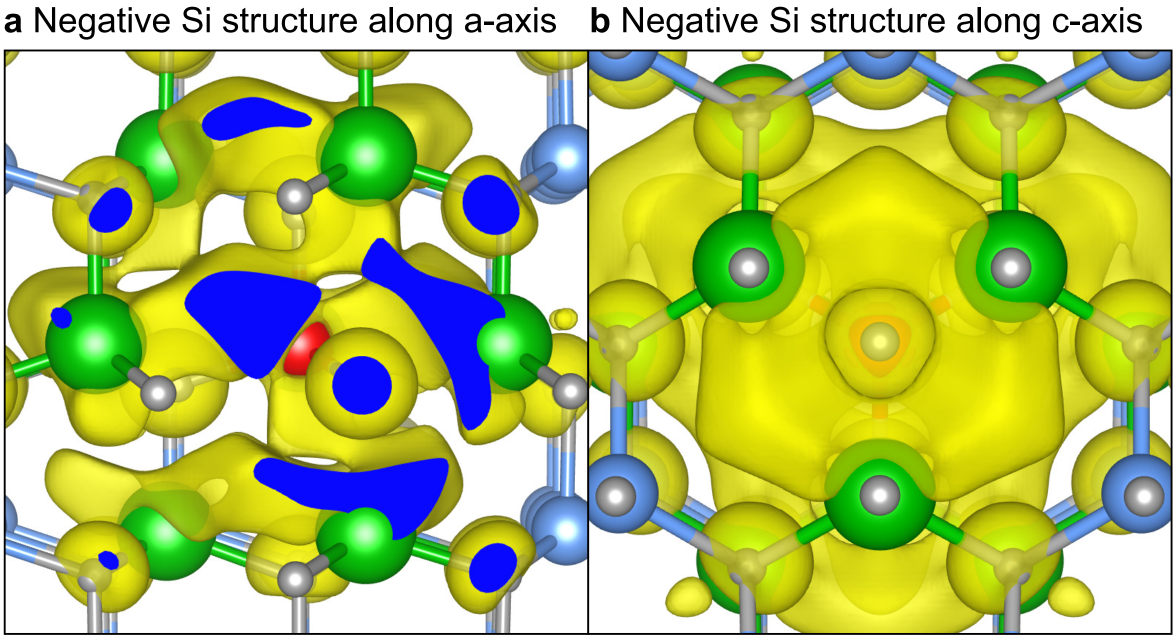

Supplementary Figure 4. Atomic structure and visualized charge density in the Si_{Al}^{-} configuration along **(a)** a- and **(b)** c-axis. The silicon atom (red sphere) sits on the substitutional site normally occupied by aluminum (blue spheres) inside a cluster formed by 12 gallium atoms (large green spheres); nitrogen atoms are denoted by small gray spheres. Yellow isosurface shows delocalized electron density of state with 5% of the maximum.

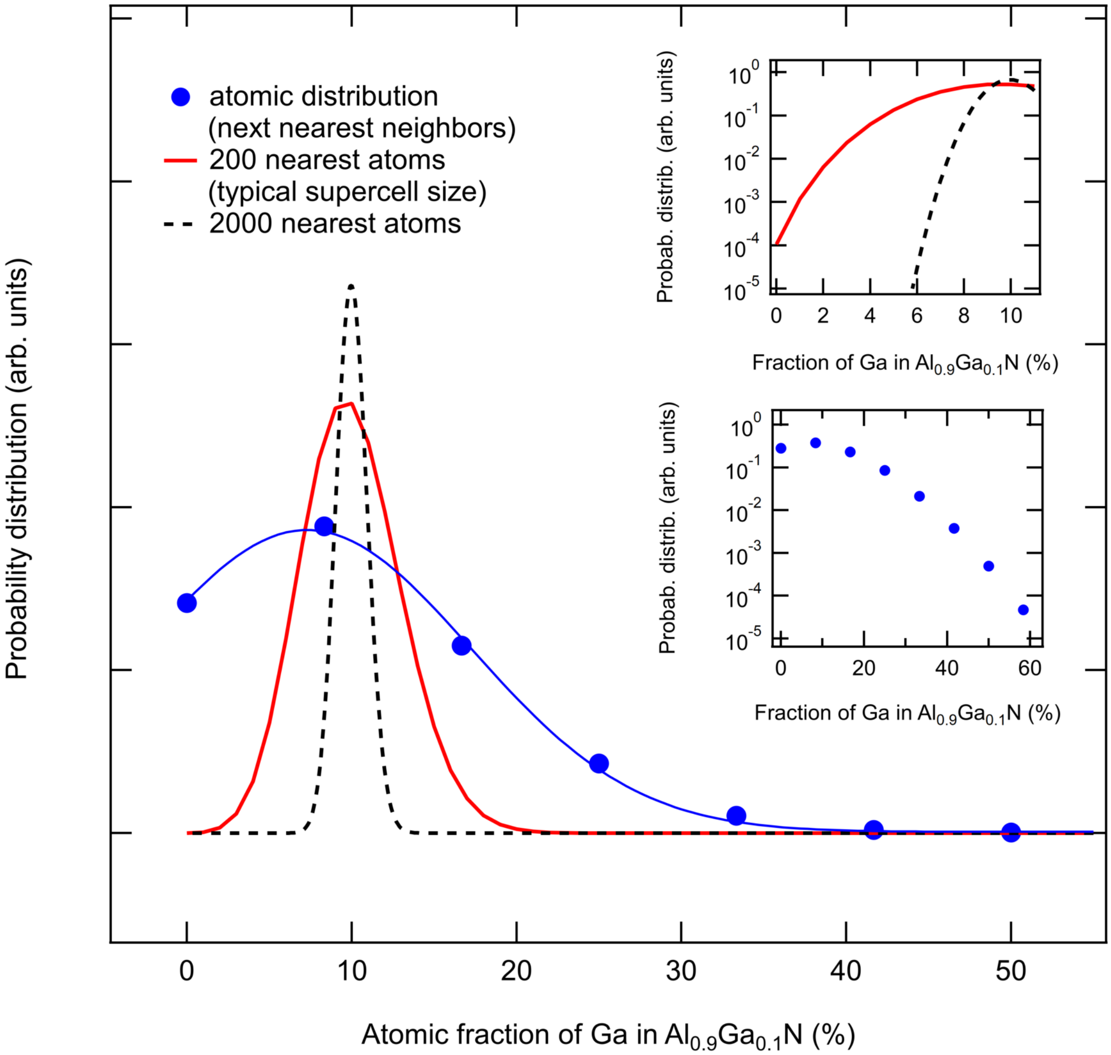

Supplementary Figure 5. Probability distributions of Ga/Al atomic arrangements in $Al_{0.90}Ga_{0.10}N$. Note that the vertical axes of the insets that show the zoomed-in ranges are on the logarithmic scale.

SUPPLEMENTARY REFERENCES

[1]J. Oila, V. Ranki, J. Kivioja, K. Saarinen, P. Hautojärvi, J. Likonen, J. M. Baranowski, K. Pakula, T. Suski, M. Leszczynski, and I. Grzegory, "Influence of dopants and substrate material on the formation of Ga vacancies in epitaxial GaN layers," Physical Review B **63**, 045205 (2001).

[2]I. Prozheev, F. Mehnke, T. Wernicke, M. Kneissl, and F. Tuomisto, "Electrical compensation and cation vacancies in Al rich Si-doped AlGaN," Applied Physics Letters **117**, 142103 (2020).

[3]J.-M. Mäki, I. Makkonen, F. Tuomisto, A. Karjalainen, S. Suihkonen, J. Räisänen, T. Y. Chemekova, and Y. N. Makarov, "Identification of the V_{Al}-$\mathrm{O_N}$ defect complex in AlN single crystals," Physical Review B **84**, 081204 (2011).

[4]B. Ravel and M. Newville, "ATHENA, ARTEMIS, HEPHAESTUS: data analysis for X-ray absorption spectroscopy using IFEFFIT," Journal of Synchrotron Radiation **12**, 537–541 (2005).

[5]S. Calvin, *XAFS for Everyone* (CRC press, 2013).

[6]D. J. Chadi and K. J. Chang, "Theory of the Atomic and Electronic Structure of DX Centers in GaAs and $\mathrm{Al}_x\mathrm{Ga}_{1-x}\mathrm{As}$ Alloys," Phys. Rev. Lett. **61**, 873–876 (1988).

[7]C. G. Van de Walle and J. Neugebauer, "First-principles calculations for defects and impurities: Applications to III-nitrides," Journal of Applied Physics **95**, 3851–3879 (2004).

[8]L. Gordon, J. L. Lyons, A. Janotti, and C. G. Van de Walle, "Hybrid functional calculations of *DX* centers in AlN and GaN," Physical Review B **89**, 085204 (2014).
